# Chikungunya Virus and *Aedes* Mosquitoes: Saliva Is Infectious as soon as Two Days after Oral Infection

**DOI:** 10.1371/journal.pone.0005895

**Published:** 2009-06-12

**Authors:** Mathieu Dubrulle, Laurence Mousson, Sara Moutailler, Marie Vazeille, Anna-Bella Failloux

**Affiliations:** Institut Pasteur, Génétique moléculaire des Bunyavirus, Paris, France; University of Liverpool, United Kingdom

## Abstract

**Background:**

*Aedes aegypti* and *Aedes albopictus* are potential vectors of chikungunya virus (CHIKV). The recent CHIKV outbreaks were caused by a new variant characterized by a mutation in the E1 glycoprotein gene (E1-226V) which has favored a better transmissibility by *Ae. albopictus*. As *Ae. albopictus* tends to replace *Ae. aegypti* in many regions, one question remained: is *Ae. albopictus* as efficient as *Ae. aegypti* to transmit the variant E1-226V of CHIKV?

**Methodology and Findings:**

We infected orally both species with the variant E1-226V and estimated the infection, the viral dissemination, and the transmission rate by real time RT-PCR. Additionally, we used an *in vitro* assay to determine the amount of virus delivered by mosquitoes in their saliva. We found that *Ae. aegypti* as well as *Ae. albopictus* ensured a high replication of the virus which underwent an efficient dissemination as detectable in the salivary glands at day 2 post-infection (pi). Infectious CHIKV particles were delivered by salivary glands from day 2 with a maximum at day 6 pi for *Ae. albopictus* (10^3.3^ PFU) and day 7 pi for *Ae. aegypti* (10^2.5^ PFU).

**Conclusions:**

*Ae. albopictus* is slightly more efficient than *Ae. aegypti* to transmit the variant E1-226V of CHIKV. These results will help to design an efficient vector control to limit transmission as soon as the first human cases are diagnosed.

## Introduction

An explosive outbreak due to an arthropod-borne virus, chikungunya virus (CHIKV), affected for the first time in 2005–2006 numerous islands in the Indian Ocean and notably La Reunion Island where one third of the population was infected [1]. It was due to a variant of CHIKV harboring a substitution A226V in the E1 glycoprotein (E1-226V) which was demonstrated to be highly transmitted by the unusual vector *Aedes albopictus* [2], [3].

This outbreak is believed to have originated in Central/East Africa where the urban mosquito *Aedes aegypti* was the most significant vector. However, the amino-acid substitution from an alanine to a valine in the E1 glycoprotein appears to be only associated to *Ae. albopictus*. Subsequent outbreaks in Madagascar, India, Cameroon, Gabon, and Italy, were due to the variant E1-226V and transmitted by *Ae. albopictus* corroborating an adaptative mutation in response to a requirement for transmission by this vector [4]. This has increased the risk for CHIKV to extend its geographic range as *Ae. albopictus* can colonize both tropical and temperate countries and is now present in all continents.

Arboviruses infect the mosquito midgut following ingestion of a viremic blood, replicate, disseminate to the salivary glands, and emerge into saliva to be transmitted when the mosquito bites. The midgut and salivary glands act as barriers to virus infection and escape [5]. The transmission of the virus to the vertebrate host depends upon the secretion of infectious virions in the saliva of the vector; the delay between feeding and being infectious is known as the extrinsic incubation period (EIP). Estimation of the amount of infectious particles transmitted by the mosquito after a blood meal is crucial to understand transmission and pathogenesis [6], [7]. Mosquito must salivate during blood feeding as the saliva contains different substances counteracting the host hemostatic response preventing blood coagulation and enhancing vasodilatation [8], [9]. However, components of saliva may differ from one species to another [10]. It has also been reported that saliva is able to enhance viral infections [11].

By histological examination and quantitative RT-PCR, CHIKV was found in the salivary glands of *Ae. albopictus* two days after ingestion of the infectious blood meal [3]. However, a question remained: were infectious viral particles present in the saliva excreted by the mosquito female at that time. For such purpose, we infected orally both *Ae. albopictus* and *Ae. aegypti* with the variant E1-226V of CHIKV, and collected saliva from day 1 to day 14 pi (post-infection) to detect the presence of infectious particles. *Ae. aegypti* was also tested in our study since it is the major vector of CHIKV in Asia [12], [13].

## Materials and Methods

### Mosquitoes

Laboratory-reared *Ae. albopictus* and *Ae. aegypti* were obtained from the DRASS (Direction Régionale des Affaires Sanitaires et Sociales) in La Reunion Island. *Ae. albopictus* Providence (ALPROV) was collected in 2006 from La Reunion Island and *Ae. aegypti* Petite-Terre (AAPT) in 2006 from Mayotte in the Comoros archipelago. Both *Ae. aegypti* and *Ae. albopictus* were present in Mayotte where the former species has been suspected to play the major role in CHIKV transmission. However, in La Reunion Island, *Ae. albopictus* acted as the main CHIKV vector [3], [14]. The F4 generation of *Ae. albopictus* and the F6 generation of *Ae. aegypti* were used for oral infections. Colonies were maintained at 28±1°C with a light∶dark cycle of 16 h∶8 h and a 80% relative humidity. Larvae were reared in pans containing 1 yeast tablet in 1 liter of tap water. Adults were provided with 10% sucrose solution *ad libitum* and fed three times a week on anaesthetized mice (OF1 mice obtained from Charles River laboratories, France). All experiments on live vertebrates were performed in compliance with French and European regulation and according to the Institut Pasteur guidelines for laboratory animal husbandry and care.

### Virus

The CHIKV 06.21 isolated in November 2005 from a new-born male from La Reunion presenting meningo-encephalitis symptoms [2] was used for all experiments. This strain contained the change A→V at the position 226 in the E1 glycoprotein (E1-226V). Stock virus was produced following three passages on *Ae. albopictus* C6/36 cells then harvested and stored at −80°C in aliquots. Procedure for C6/36 cell infections and passages are described elsewhere [3]. The titer of the frozen stock virus was estimated to 10^9^ plaque-forming units (PFU)/mL.

### Experimental oral infections

Blood meals were prepared as follows: 1 mL of viral suspension was added to 2 mL of washed rabbit erythrocytes supplemented with ATP (5×10^−3^ M) as a phagostimulant. The infectious blood at a titer of 10^7.5^ PFU/mL was transferred in a glass feeder maintained at 37°C and placed on top of the mesh of a plastic box containing 60 females of 1-week-old that had been starved for 24 hours prior to the infection experiment. After 15 min of feeding, engorged females were sorted on ice and transferred to cardboard containers. Females were fed with 10% sucrose at 28°C. The entire feeding period lasted one hour with no significant change in the viral titer.

### Female status analyzed by IFA

Females were sacrified and tested for the presence of CHIKV on head squashes by immunofluorescence assay (IFA) [15]. CHIKV antigen was detected with a mouse ascetic fluid provided by the French National Reference Center for Arbovirus of the Institut Pasteur.

### Real-time RT-PCR assays

To measure dissemination and transmission at different days after infection, five females were killed every 1–2 days until day 14 pi. Total RNA was extracted on wings, salivary glands and bodies using the Nucleospin® RNA II kit (Macherey-Nagel) following the manufacturer's protocol. The SYBR Green technology was used to quantify the amount of virus in each sample. The one-step RT-PCR was performed with a Power SYBR® Green RNA-to-CT™ one step kit (Applied Biosystem) in a volume of 25 µL containing 2 µL RNA template, 12.5 µL 2× Power SYBR® Green I RT-PCR Mix , 0.25 µL sense (2.5 µM), 0.25 µL anti-sense (2.5 µM), 0.2 µL RT enzyme mix and 9.8 µL of ddH_2_O. Primers were selected in the E2 structural protein regions of sequences retrieved from the GenBank database by the Laboratory for Urgent Response to Biological Threats at the Institut Pasteur: sense Chik/E2/9018/+ (CACCGCCGCAACTACCG) and anti-sense Chik/E2/9235/- (GATTGGTGACCGCGGCA). The amplification program in an Applied Biosystem 7700 real time PCR system included: a reverse transcription at 48°C for 30 min, an activation step of the polymerase at 95°C 10 min followed by 40 cycles of 15 s at 95°C and 1 min at 60°C, 72°C 30 s, a step at 95°C 20 s, with a final ramping of 19 min 59 sec. The size of the amplification product was 217 bp. A standard curve was generated using duplicates of 10-fold serial dilutions of RNA synthetic transcripts (for more details, see [3]). Quantification of viral RNA was done by comparison of the threshold cycle (Ct) values of the samples to the standards according to the ΔC_t_ analysis. One assay was carried out for each mosquito species.

### Saliva collection and titration

Twenty females were used every 1–2 days until day 14 pi. Females were chilled, and wings and legs were removed and discarded. Proboscis was inserted into 1 µL micropipette (microcaps®, Drummond Scientific Company, PA, USA) filled with 1 µL of Fetal Bovine Serum (FBS). One µL of 1% pilocarpine, an analogue of the acetylcholine, prepared in PBS at 0.1% Tween 80, was applied on the thorax to stimulate salivation. After 45 min, medium containing the saliva was expelled under pressure into 1.5 mL tubes containing 29 µL of DMEM (Dulbecco's modified Eagle's medium) supplemented with 10% FBS. These 30 µL were added, diluted or undiluted, to monolayers of Vero cells to detect infectious particles by the plaque assay technique. Cells were incubated for 3 days at 37°C under an overlay consisting of DMEM 2×, 2% FBS, antibiotics and 1% Indubiose (IBF Biotechnics). Plaques were counted after staining with a solution of crystal violet (0.2% in 10% formaldehyde and 20% ethanol). The titer of infectious particles per saliva was expressed as PFU/mL. One assay was achieved for each mosquito species.

In addition to collection and titration of saliva, 5 females at different days pi were tested for the presence of CHIKV on head squashes to evaluate the relation between the presence of CHIKV in saliva and head squashes positive by IFA.

### Parameters analyzed

Three parameters describing vector competence were determined: (i) mosquito infection measured by detecting viral RNA in bodies (thorax and abdomen), (ii) viral dissemination by quantification of viral RNA in mosquito wings, and (iii) transmission potential by measuring viral RNA in salivary glands and excreted saliva by plaque assay on cell cultures.

## Results

### Mosquito infection

**Figure 1:**
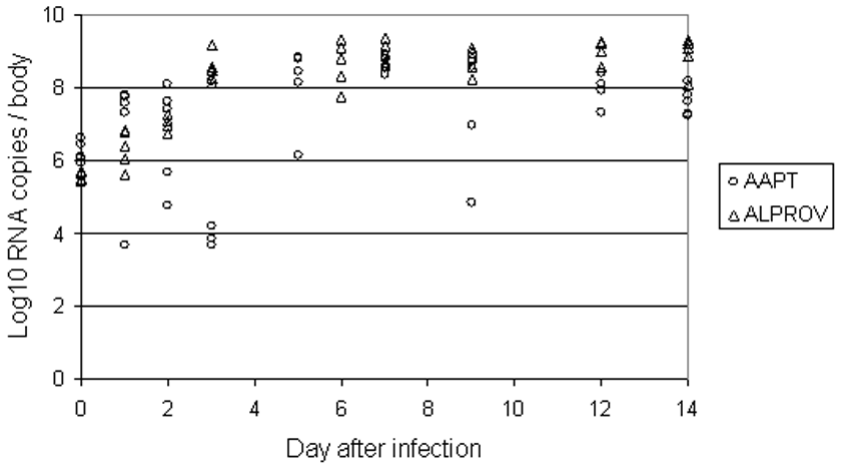
Infection of *Aedes albopictus* Providence (ALPROV) and *Aedes aegypti* Petite-Terre (AAPT) with CHIKV 06.21. At different days after exposure to the infectious blood meal, mosquito infection was measured by detecting viral RNA in bodies (thorax and abdomen) of 5 females by real-time RT-PCR.

At day 0 pi (just after the blood meal), the number of viral RNA copies in the thorax and abdomen of females ranged from 10^5.4^ to 10^5.7^ (mean±standard deviation: 10^5.6^±10^0.1^) for *Ae. albopictus* and from 10^5.9^ to 10^6.6^ (10^6.2^±10^0.3^) for *Ae. aegypti* (Figure 1). For *Ae. albopictus*, the number of copies increased until day 3 pi (10^8.1^–10^9.2^ with 10^8.5^±10^0.4^) then reached a plateau and stayed steady until day 14 pi (10^8.1^–10^9.3^ with 10^8.9^±10^0.5^). For *Ae. aegypti*, the maximum was reached at day 7 pi (10^8.3^–10^8.9^ with 10^8.6^±10^0.2^), then decreased slightly until day 14 pi (10^7.2^–10^8.2^ with 10^7.6^±10^0.4^). Values were more variable between *Ae. aegypti* females at different days until day 12 pi.

### CHIKV dissemination

The disseminated infection rate was evaluated by IFA detection of CHIKV on head squashes of females sacrified 14 days pi. For *Ae. albopictus*, disseminated infection rates varied from 91% (N = 45 surviving females) and 94.5 (55) when examining female status at day 14 pi in the two replicates. For *Ae. aegypti*, rates ranged from 88.5% (26) to 90.7% (54). No significant difference of rates was observed (Fisher's exact test: P = 0.71) between replicates and species.

**Figure 2:**
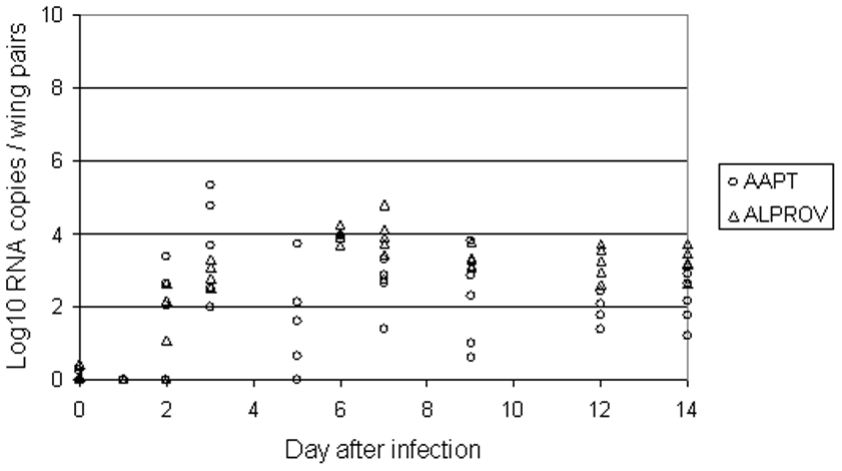
CHIKV dissemination in *Aedes albopictus* Providence (ALPROV) and *Aedes aegypti* Petite-Terre (AAPT). At different days after oral infection, viral dissemination was measured by quantification of viral RNA in mosquito wings of 5 females by real-time RT-PCR.

When examining viral dissemination inside female by RNA quantification in wings at different days after exposure to infectious meal, viral RNA started to be detectable at day 2 pi for both species: values ranged from 10^1.1^ to 10^2.7^ (10^1.1^±10^1.3^) for *Ae. albopictus* and from 10^2.0^ to 10^3.3^ (10^1.5^±10^1.6^) for *Ae. aegypti* (Figure 2). It reached a maximum (10^3.4^–10^4.8^ with 10^4^±10^0.5^) at day 7 pi for *Ae. albopictus* and at day 3 pi (10^2.0^–10^5.3^ with 10^3.7^±10^1.4^) for *Ae. aegypti*. Values were more variable between *Ae. aegypti* females at different days pi.

### CHIKV transmission

#### Analysis of salivary glands

**Figure 3:**
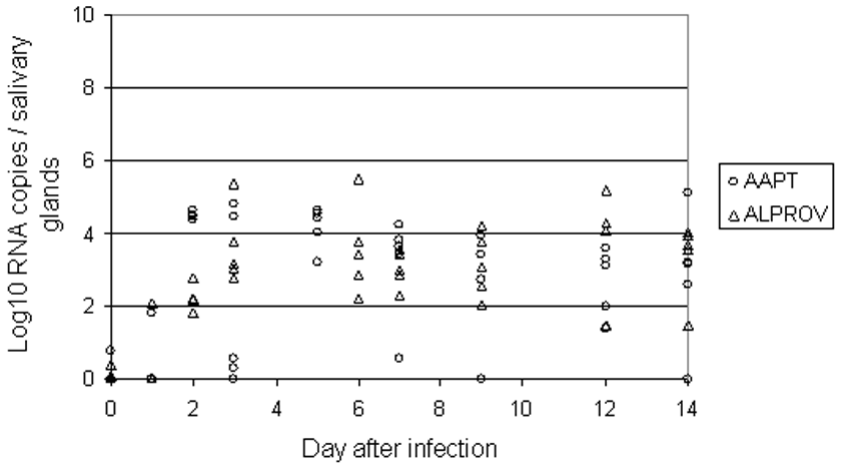
CHIKV transmission by *Aedes albopictus* Providence (ALPROV) and *Aedes aegypti* Petite-Terre (AAPT). At different days after oral infection, transmission was evaluated by detecting viral RNA in salivary glands of 5 females by real-time RT-PCR.

For both species, 1–2 individuals among five presented CHIKV-positive salivary glands at day 1 pi. Nevertheless, the majority of mosquitoes harbored viral RNA in the salivary glands from day 2 pi (Figure 3). For *Ae. albopictus*, the number of viral RNA varied from 10^1.8^ to 10^2.8^ (10^2.3^±10^4.1^) at day 2 pi and increased at day 3 pi (10^2.7^–10^5.4^ with 10^3.6^±10^1.0^) then stayed steady until to day 14 pi (10^1.5^–10^4.0^ with 10^3.3^±10^1.0^). For *Ae. aegypti*, the number of viral RNA reached a maximum at day 2 (10^4.4^–10^4.6^ with 10^4.5^±10^0.1^) followed by a plateau from day 3 pi (10^0.3^–10^4.8^ with 10^2.0^±10^2.4^) to day 14 pi (10^2.6^–10^5.1^ with 10^2.7^±10^1.9^).

#### Analysis of saliva

**Figure 4:**
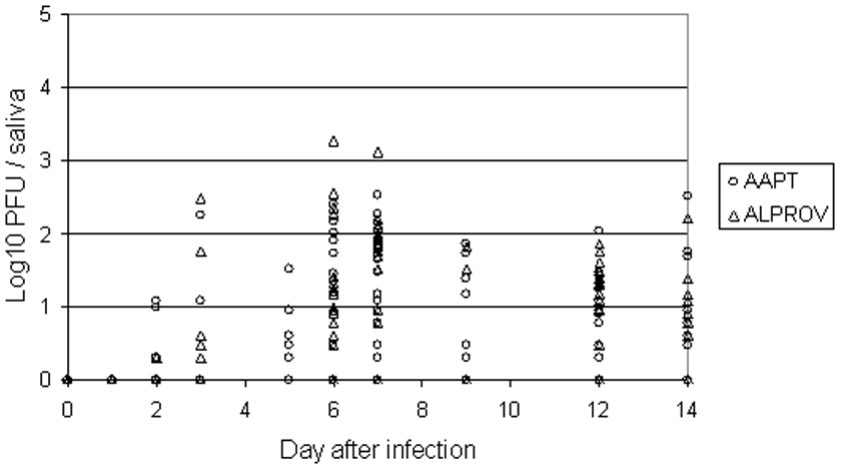
CHIKV present in saliva of *Aedes albopictus* Providence (ALPROV) and *Aedes aegypti* Petite-Terre (AAPT). At different days after oral infection, transmission potential was assessed by estimating the number of infectious viral particles in excreted saliva by plaque assay on cell cultures.

For *Ae. albopictus* (Figure 4), infectious CHIKV particles started to be detectable in the saliva at day 2 pi (one individual with 10^0.3^ PFU). The maximum was reached between day 6 pi (10^0.5^–10^3.3^ with 10^1.4^±10^0.7^) and day 7 pi (10^0.8^–10^3.1^ with 10^1.8^±10^0.5^). For *Ae. aegypti* (Figure 4), CHIKV were present in the saliva at day 2 pi (four individuals with 10^0.8^±10^0.4^). The maximum of viral particles was reached between day 6 pi (10^0.5^–10^2.4^ with 10^1.6^±10^0.7^) and day 7 pi (10^0.3^–10^2.5^ with 10^1.2^±10^0.7^).

#### Relation between the presence of infectious particles in saliva and IFA-positive head squashes

**Figure 5:**
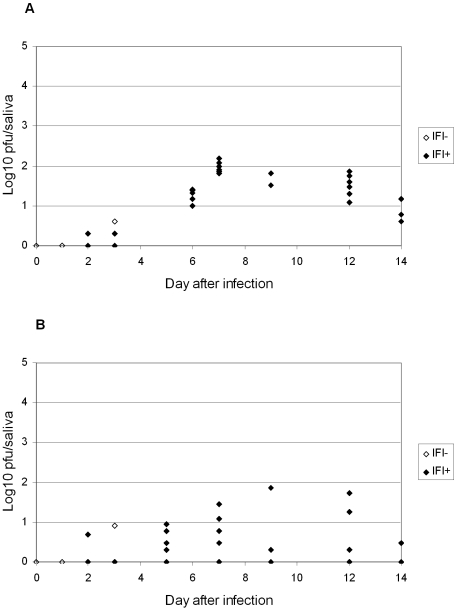
Relation between CHIKV in saliva and IFA-positive head squashes of (A) ALPROV and (B) AAPT. At different days after oral infection, 5 females were tested for the presence of CHIKV on head squashes and the number of infectious viral particles in excreted saliva to evaluate the relation between the presence of CHIKV in saliva and head squashes positive by IFA.

For *Ae. albopictus* (Figure 5A) and *Ae. aegypti* (Figure 5B), head squashes were CHIKV-positive with females able to deliver virus through saliva from day 2 pi. These results corroborate a significant relation between the presence of the virus in the head and the ability of the female to excrete virus by saliva. However, *Ae. albopictus* exhibit more homogeneous results.

## Discussion

This report describes the first analysis of CHIKV in the saliva of *Ae. albopictus* and *Ae. aegypti*. Viral infectious particles were detectable in the saliva two days after ingestion of the infectious blood meal. Moreover, dissemination within the mosquito was very rapid and concomitant to colonization of salivary glands. Compared to *Ae. aegypti*, the number of infectious particles in saliva was slightly higher in *Ae. albopictus* confirming the role of *Ae. albopictus* as an efficient epidemic CHIKV vector during La Reunion outbreak. It should be stressed that despite the fact that *Ae. albopictus* females took smaller infectious meals (i.e. less infectious particles) in our artificial feeding system, this species was able to deliver more infectious particles in its saliva.

Mosquito populations differ in their susceptibility to ensure viral replication. The vector competence reflects the different barriers encountered by the virus from its entry into the mosquito midgut to its release in the saliva. After penetrating the midgut cells, the virus replicates and disseminates to the haemocel and subsequently, to other organs. The timing of dissemination is quite variable but once virus has entered the haemocel, numerous tissues can be infected quickly illustrating the midgut as a barrier to the virus entry. A dose-response phenomenon described as the minimum threshold for infection characterizes each vector-virus combination. During the course of an infection, the titer of the virus in the mosquito changes: in the early stage of infection, an eclipse phase can occur before the virus actively replicates in the midgut [16]. It has not been observed in our study (see Figure 1). Typically dissemination occurs only after replication within the mesenteron, although the efficiency of dissemination may be influenced by dose [3]. Once the infectious particles have been released in the haemocel, the salivary glands must be considered as the most important target organ. To infect these glands, virus must pass through the outer basement membrane on which the salivary acinar cells rest, enter the cell and then replicate within the cytoplasm. The virus then must be shed into the saliva to be transmitted during feeding. The EIP is very relevant epidemiologically: it has been found to be 2 days for Rift Valley fever virus in *Culex pipiens* [17] and for Venezuelan Equine Encephalitis virus in *Ae. aegypti* [18]. We also found similar data: CHIKV is present in saliva from day 2 after ingestion of an infectious blood meal. A short EIP would have important consequences on CHIKV transmission.

Transmission of arboviruses from a vector to a vertebrate host typically occurs when saliva is expectorated during the process of blood feeding. Both species were able to be highly infected, *Ae. aegypti* ingesting a higher quantity of virus. The larger size of this species would be one explanation [19]. Nevertheless, *Ae. albopictus* ensured a higher rate of replication as the estimated number of viral RNA increased nearly four fold from the ingestion to the maximum reached at day 3 pi. Dissemination examined by quantifying viral RNA in wings began from day 2 pi and reached a maximum at day 3 pi for *Ae. aegypti* and later at day 7 pi for *Ae. albopictus*. The virus was detectable in the salivary glands from day 2 pi, reached a maximum at day 3 pi then stayed stable until day 14 pi for both species. Concomitantly, the two vectors were able to deliver saliva with infectious particles from day 2 pi. Thus the virus was excreted as soon as it has been produced in the salivary glands. By using direct dilution of excreted saliva, we were able to perform titration on Vero cells without filtration, which is impractical for such small volumes. We found that *Ae. albopictus* infected with the variant E1-226V was capable to transmit an average of 1 viral particle from day 1–2 after ingestion of the infectious meal with a maximum at day 6 pi (10^3.3^ particles). Besides, *Ae. aegypti* was able to deliver an average of 1–2 virus particles from day 2 pi with a maximum at day 6 (10^2.4^ particles) or day 7 pi (10^2.5^ particles). It is known that the saliva is flushed out just after immersing the mosquito proboscis in fluid; there is no reservoir for saliva except the lumen of the ducts which probably would not contain more than 0.1 nL [20]. Mosquitoes such as *Ae. aegypti* salivate an average of 4.7 nL during the blood feeding process [21] but the amount of viral particles transmitted by a vector is highly variable depending on the technique used for detection [6], [7], [22], [23], [24]. The amount of virus inoculated by a mosquito while feeding on a live host could be ∼600 fold higher than that recovered during an *in vitro* capillary tube assay [25]. A more accurate technique could be to allow mosquitoes to feed on hanging blood drops [26].

In nature, *Ae. albopictus* as well as *Ae. aegypti* feed frequently and almost exclusively on humans. In addition, these anthropophilic mosquitoes may ingest 2–3 blood-meals during a single gonotrophic cycle [27], [28] and when infected with an arbovirus, are able to transmit the virus for life. Thus these two mosquito species with a short EIP, persistently infected salivary glands and the ability to feed several times within a gonotrophic cycle, would have an invaluable epidemic potential to transmit CHIKV. Control efforts should therefore be initiated rapidly and maintained to target mosquitoes which are able to transmit two days after ingestion of an infectious blood meal from a viremic host.
